# Supplementary material for: Evaluation of community pharmacists’ knowledge and attitude about Hajj and Umrah-related health conditions in the western region, Saudi Arabia: A cross-sectional study
Source: PLoS One. 2025 Mar 18;20(3):e0317000. doi: 10.1371/journal.pone.0317000 (PMC11918331; doi:10.1371/journal.pone.0317000)
Supplement: Attachment — Submitted filename: Response letter.pdf [file pone.0317000.s003.pdf]

PONE-D-24-02546

Evaluation of Community Pharmacists' Knowledge and Attitude about Hajj and Umrah-related health conditions in the Western Region, Saudi Arabia: A Cross-sectional study.

Dear Mohamed R. Abonazel, Ph.D.

Academic Editor

PLOS ONE

Comments to the Author

1. Is the manuscript technically sound, and do the data support the conclusions?

The manuscript must describe a technically sound piece of scientific research with data that supports the conclusions. Experiments must have been conducted rigorously, with appropriate controls, replication, and sample sizes. The conclusions must be drawn appropriately based on the data presented.

Reviewer #1: Partly

Reviewer #2: Yes

Author response: Thanks indeed.

2. Has the statistical analysis been performed appropriately and rigorously?

Reviewer #1: No

Reviewer #2: Yes

Author response: Thanks indeed. We made the requested changes.

3. Have the authors made all data underlying the findings in their manuscript fully available?

Reviewer #1: Yes

Reviewer #2: Yes

Author response: Thanks indeed for your positive feedback.

4. Is the manuscript presented in an intelligible fashion and written in standard English?

Reviewer #1: Yes

Reviewer #2: Yes

Author response: Thanks indeed for your positive feedback.

5. Review Comments to the Author

Reviewer #1: 1. Author need to do statistical analyses (significance level of p ;0.05)

2. How the author makes sure that the participants were not biased towards specific answer

3. How the author makes sure that the participants were not disengaged while answering.

4. How the author makes sure about reliability of result

Author response: The study implemented rigorous measures to mitigate potential predispositions. Randomized assignment was employed to distribute participants evenly across conditions, minimizing selection bias. The design of the questionnaire was carefully crafted to avoid leading or suggestive language, and a pre-testing phase was conducted to identify and rectify any potential biases from community pharmacists in western region of Saudi Arabia. Additionally, blinding techniques were incorporated to ensure both participants and experimenters remained unaware of specific conditions, reducing the risk of bias stemming from expectations. The study also prioritized diverse participant demographics to enhance external validity because mostly of the community pharmacists are non-Saudis. Lastly, the importance of informed consent and debriefing procedures was emphasized, ensuring participants were adequately informed about the study's nature and potential biases. These comprehensive strategies collectively contribute to bolstering the study's internal validity and minimizing the likelihood of participant bias towards specific answers. Significance level of p ;0.05 were applied for all.

Reviewer #2:

It is an honor for me to review a manuscript number PONE-D-24-02546; Evaluation of Community Pharmacists; Knowledge and Attitude about Hajj and Umrah-related health conditions in the Western Region, Saudi Arabia: A Cross-sectional Study. The manuscript looks interesting and significant locally however, the following major modification is required.

The introduction is poorly described and not aligned with the study objective.

Author response: Thanks indeed. We updated the whole manuscript including the introduction.

Kindly add the rationale of the study under the last paragraph of the introduction

Author response: Thanks indeed. Done.

Kindly add how survey tools were developed

Author response: Thanks indeed. The tool was explained under ‘’ Questionnaire design’ section.

Kindly write Cronbach’s alpha values

Author response: Thanks indeed.

Kindly add how survey respondents were selected.

Author response: Thanks indeed. We have updated the whole paragraph under ‘’ 2.2 Survey respondent selection process and data collection.’’

How survey link was disseminated among the respondents

Author response: Thank you for your insightful question. The survey link was disseminated among the respondents through a google form link. Specifically, we utilized email invitations, institutional mailing lists, etc.] to reach our target audience.

Kindly add the study site

Author response: Thanks indeed. We added the study site. Community pharmacists (CPs) living Makkah, Madinah, Jeddah and Taif which represent in the western region of Saudi Arabia

How study respondents were biased

Author response: Thanks indeed. The study implemented rigorous measures to mitigate potential predispositions. Randomized assignment was employed to distribute participants evenly across conditions, minimizing selection bias. The design of the questionnaire was carefully crafted to avoid leading or suggestive language, and a pre-testing phase was conducted to identify and rectify any potential biases from community pharmacists in western region of Saudi Arabia. Additionally, blinding techniques were incorporated to ensure both participants and experimenters remained unaware of specific conditions, reducing the risk of bias stemming from expectations. The study also prioritized diverse participant demographics to enhance external validity because mostly of the community pharmacists are non-Saudis. Lastly, the importance of informed consent and debriefing procedures was emphasized, ensuring participants were adequately informed about the study's nature and potential biases. These comprehensive strategies collectively contribute to bolstering the study's internal validity and minimizing the likelihood of participant bias towards specific answers.

Kindly add exclusion criteria for the study

Author response: Thanks indeed. Done as of ‘’The study excluded CPs from the other regions of Saudi Arabia and those who are not in the practice’’.

Discussion needs to update and compare findings with the latest published article

Author response: Thank you for your constructive feedback. We appreciate your suggestion to update and compare our findings with the latest published articles. However, the limited availability of relevant literature in for Hajj and Umrah poses a challenge. We have thoroughly searched for recent publications and, while the number is limited, we have incorporated the most pertinent ones into the discussion. We hope this addresses your concern, and we appreciate your understanding of the constraints in our field.

Please scan your manuscript again and correct some topological errors.

Author response: Thanks indeed. The manuscript underwent a significant English proofreading, and you can check the tracking.

Please make sure your manuscript was written in line with this journal submission guideline as well as reference styles

Author response: Thanks indeed. Done.

6. PLOS authors have the option to publish the peer review history of their article (what does this mean?). If published, this will include your full peer review and any attached files.

Do you want your identity to be public for this peer review? For information about this choice, including consent withdrawal, please see our Privacy Policy.

Reviewer #1: No

Reviewer #2: No
