# Supplementary material for: Evaluation of community pharmacists’ knowledge and attitude about Hajj and Umrah-related health conditions in the western region, Saudi Arabia: A cross-sectional study
Source: PLoS One. 2025 Mar 18;20(3):e0317000. doi: 10.1371/journal.pone.0317000 (PMC11918331; doi:10.1371/journal.pone.0317000)
Supplement: Attachment — Submitted filename: Response Letter 03.12.2024.pdf [file pone.0317000.s005.pdf]

PONE-D-24-02546

3rd December 2024

Evaluation of Community Pharmacists; Knowledge and Attitude about Hajj and Umrah-related health conditions in the Western Region, Saudi Arabia: A Cross-sectional study.

Dear Mohamed R. Abonazel, Ph.D.

Academic Editor

PLOS ONE

Comments to the Author

Reviewer #1:

1. Result of time spend on providing Hajj and Umrah health related conditions advice pilgrims is non-significant. How the author ensures the comparison of this study with another study.

Thank you for your valuable comment. The study acknowledges the non-significant results regarding the time spent providing health advice to pilgrims. To ensure comparability with other studies, we carefully considered the following:

1. **Study Design and Population:** Our methodology aligns closely with similar studies conducted in Hajj and Umrah contexts. The selection criteria for participants and the health conditions addressed were standardized based on guidelines commonly used in comparable research.
2. **Outcome Measures:** The key performance indicators, such as time spent on consultations and the specific health advice given, were selected to mirror metrics from previous studies, facilitating a meaningful comparison.
3. **Contextual Factors:** We accounted for unique contextual factors such as the dynamic and transient nature of Hajj and Umrah pilgrimages, which may differ from the settings in other studies. This context was clearly stated as a potential limitation.

2. How the author makes sure that the participants were not biased towards specific answer

Response: Thank you for your comment. We appreciate your concern and agree that self-reported questions may be biased. Therefore, we have added a statement addressing this issue. The use of self-reported questionnaires may introduce social desirability bias in the responses provided. As a result, future

studies utilizing other data collection methods are necessary to ensure more accurate responses.

3. How the author makes sure that the participants were not disengaged while answering.

Response: Thank you for your comment. We appreciate your concern. The data was collected using online Google Form questionnaires. The author did not know the participants' status during the questionnaire responses. However, we have mentioned this as a limitation.

4. Author need to mention response rate

Response: It was added

Reviewer #2: All the raised comments have been rectified by the comments. However, I am still worried about sampling and sample size calculator proper citation. Kindly add a suitable reference for sampling and a sample size calculation. Rather than just link OpenEpi: Sample Size for X-Sectional, Cohort,and Clinical Trials . However, you may use this reference for sampling and a sample size calculation.

Response: Thank you for the comment. We appreciate your suggestion, and the references have been added.

Ali MD, Hatef EAJA. Types of Sampling and Sample Size Determination in Health and Social Science Research. Journal of Young Pharmacists [Internet]. 2024 Jun 6;16(2):204–15. Available from: http://dx.doi.org/10.5530/jyp.2024.16.27 and cite them properly.

Reviewer #3: 1. The "Conclusion" section needs improvement.

2. The paper contains a few grammatical errors. The authors need to review the full text carefully.

Response: Thank you for the comment. The entire manuscript has been revised for English.

3. Recently published papers are relevant to this manuscript, so one or all of them should be cited, such as

doi: https://doi.org/10.1016/j.jth.2022.101353 - https://doi.org/10.1371/journal.pone.0250149

Response: We appreciate your insightful feedback. We have included additional literature by citing the references you suggested.
